# (1*R*,1′*R*,3*S*,3′*S*)-5,5′,10,10′-Tetra­meth­oxy-1,1′,3,3′-tetra­methyl-3,3′,4,4′-tetra­hydro-1*H*,1′*H*-8,8′-bi[benzo[*g*]isochromene]

**DOI:** 10.1107/S1600536808007599

**Published:** 2008-03-29

**Authors:** Jimmy J. P. Sejberg, Jonathan Sperry, Ka Wai Choi, Peter D. W. Boyd, Margaret A. Brimble

**Affiliations:** a Department of Chemistry, University of Auckland, Private Bag 92019, Auckland, New Zealand

## Abstract

In the title compound, C_34_H_38_O_6_, the methyl groups on each pyran ring exhibit 1,3-*cis* stereochemistry, established during synthesis by pseudo-axial delivery of hydride during a lactol reduction step. In the crystal structure, the mol­ecule lies on a twofold rotation axis and the torsion angle about the central diaryl bond is 41.3 (1)°. The mol­ecules pack in a herringbone arrangement.

## Related literature

For details of the synthesis, see: Brimble *et al.* (2008 ▶). For related literature, see: Brenstrum *et al.* (2001 ▶); Gibson *et al.* (2007 ▶); Gill *et al.* (1997*a* ▶,*b* ▶).
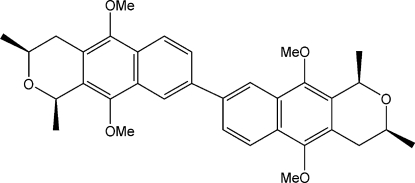


## Experimental

### 

#### Crystal data

C_34_H_38_O_6_
*M* *_r_* = 542.64
Orthorhombic, 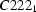

*a* = 8.8773 (2) Å
*b* = 13.9298 (2) Å
*c* = 23.1234 (4) Å
*V* = 2859.42 (9) Å^3^
*Z* = 4
Mo *K*α radiation
μ = 0.09 mm^−1^
*T* = 89 (2) K
0.36 × 0.28 × 0.22 mm

#### Data collection

Siemens SMART CCD diffractometer
Absorption correction: multi-scan (*SADABS*; Bruker, 2001 ▶) *T*_min_ = 0.889, *T*_max_ = 0.981
22789 measured reflections
2035 independent reflections
1542 reflections with *I* > 2σ(*I*)
*R*_int_ = 0.052

#### Refinement

*R*[*F* ^2^ > 2σ(*F* ^2^)] = 0.040
*wR*(*F* ^2^) = 0.095
*S* = 1.13
2035 reflections
185 parameters
H-atom parameters constrained
Δρ_max_ = 0.21 e Å^−3^
Δρ_min_ = −0.19 e Å^−3^

### 

Data collection: *SMART* (Siemens, 1995 ▶); cell refinement: *SAINT* (Siemens, 1995 ▶); data reduction: *SAINT*; program(s) used to solve structure: *SHELXS97* (Sheldrick, 2008 ▶); program(s) used to refine structure: *SHELXL97* (Sheldrick, 2008 ▶); molecular graphics: *ORTEPIII* (Burnett & Johnson, 1996 ▶) and *Mercury* (Macrae *et al.*, 2006 ▶); software used to prepare material for publication: *publCIF* (Westrip, 2008 ▶).

## Supplementary Material

Crystal structure: contains datablocks I, global. DOI: 10.1107/S1600536808007599/bi2283sup1.cif

Structure factors: contains datablocks I. DOI: 10.1107/S1600536808007599/bi2283Isup2.hkl

Additional supplementary materials:  crystallographic information; 3D view; checkCIF report

## Figures and Tables

**Fig. 1.**
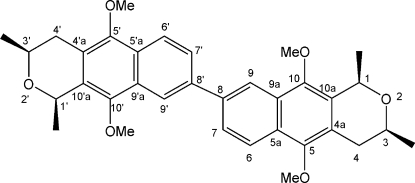
Scheme showing the standard chemical numbering scheme.

**Fig. 2.**
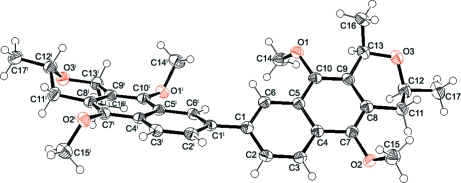
The molecular structure with displacement ellipsoids drawn at the 50% probability level for non-H atoms, showing the crystallographic numbering scheme. Symmetry code: (i) x, -y, 1 - z.

**Fig. 3.**
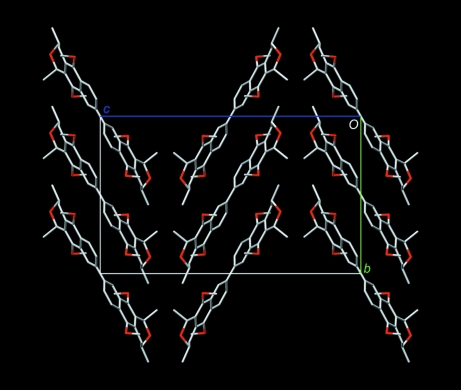
Molecular packing viewed down the a-axis, with H atoms omitted.

## supplementary crystallographic information

### Comment

Recent synthetic effort has been directed towards enantioselective synthesis of the dimeric pyranonaphthoquinone core of the cardinalins which were isolated from the New Zealand toadstool *Dermocybe cardinalis* (Gill *et al.*, 1997*a*, 1997*b*). We now report the crystal structure of the title compound (Fig. 1). The assignment of absolute stereochemistry is based on the initial use of a chiral pool reagent in the synthetic sequence. Since the stereochemistry at C3 in the pyran rings (C12 in the crystallographic numbering scheme; Fig. 2) is known to be *S*, the absolute configuration at C1 (C13 in the crystallographic numbering scheme) has therefore been assigned as *R*. The torsion angle about the diaryl bond is 41.3 (1)°. The molecules pack in a herringbone arrangement (Fig. 3).

### Experimental

To a solution of 1,1'-(6,6'-*bis*((*S*)-2-(*tert*-butyldiphenylsilyloxy)propyl)-5,5',8,8'-tetramethoxy-2,2'-binaphthyl-7,7'-diyl)diethanone (144 mg, 0.14 mmol) in THF (5 ml) was added a 1 *M* solution of tetra-*n*-butylammonium fluoride (3.0 ml, 3.0 mmol). The reaction mixture was stirred under nitrogen at room temperature for 3 d then concentrated *in vacuo*. The resulting residue was flushed through a pad of silica (hexanes-ethyl acetate 1:1–1:3). The filtrate was concentrated *in vacuo* and the resulting oil was dissolved in distilled dichloromethane (5 ml) and cooled to 195.15 K. Trifluoroacetic acid (0.065 ml, 0.86 mmol) was added and the reaction mixture was stirred for 15 min before addition of triethylsilane (0.13 ml, 0.80 mmol). The reaction mixture was then allowed to reach room temperature over 16 h. Water (20 ml) was added and the mixture extracted with ethyl acetate (20 ml × 3). The combined organic extracts were dried over anhydrous magnesium sulfate, filtered, concentrated *in vacuo* and the resulting residue was purified by flash chromatography eluting with hexanes-ethyl acetate (7:3) to give the title compound (52 mg, 0.096 mmol, 70%) as a pale yellow solid which was recrystallized from diethyl ether-dichloromethane; m.p. 541.15–542.15 K

### Refinement

H atoms were placed in calculated positions and were refined using a riding model (C–H = 0.93 or 0.97 Å), with *U *_iso_(H) = 1.2 or 1.5 times *U*_eq_(C). In the absence of significant anomalous scattering, the absolute configuration could not be determined and Friedel pairs were merged. The configuration was inferred from the known stereochemistry (*S*) of C12.

### Crystal data

**Table:**

C_34_H_38_O_6_	*D*_x_ = 1.261 Mg m^−^^3^
*M**_r_* = 542.64	Melting point: 541.15 K
Orthorhombic, *C*222_1_	Mo *K*α radiation λ = 0.71073 Å
Hall symbol: C 2c 2	Cell parameters from 4238 reflections
*a* = 8.8773 (2) Å	θ = 1.8–28.6º
*b* = 13.9298 (2) Å	µ = 0.09 mm^−^^1^
*c* = 23.1234 (4) Å	*T* = 89 (2) K
*V* = 2859.42 (9) Å^3^	Needle, pale yellow
*Z* = 4	0.36 × 0.28 × 0.22 mm
*F*_000_ = 1160	

### Data collection

**Table:**

Siemens SMART CCD diffractometer	2035 independent reflections
Radiation source: fine-focus sealed tube	1542 reflections with *I* > 2σ(*I*)
Monochromator: graphite	*R*_int_ = 0.052
*T* = 89(2) K	θ_max_ = 28.6º
ω scans	θ_min_ = 1.8º
Absorption correction: multi-scan(SADABS; Bruker, 2001)	*h* = −11→11
*T*_min_ = 0.889, *T*_max_ = 0.981	*k* = −18→18
22789 measured reflections	*l* = −27→30

### Refinement

**Table:**

Refinement on *F*^2^	Secondary atom site location: difference Fourier map
Least-squares matrix: full	Hydrogen site location: inferred from neighbouring sites
*R*[*F*^2^ > 2σ(*F*^2^)] = 0.040	H-atom parameters constrained
*wR*(*F*^2^) = 0.095	*w* = 1/[σ^2^(*F*_o_^2^) + (0.0484*P*)^2^ + 0.1354*P*] where *P* = (*F*_o_^2^ + 2*F*_c_^2^)/3
*S* = 1.13	(Δ/σ)_max_ < 0.001
2035 reflections	Δρ_max_ = 0.21 e Å^−^^3^
185 parameters	Δρ_min_ = −0.19 e Å^−^^3^
Primary atom site location: structure-invariant direct methods	Extinction correction: none

### Special details

**Table:**

Geometry. All e.s.d.'s (except the e.s.d. in the dihedral angle between two l.s. planes) are estimated using the full covariance matrix. The cell e.s.d.'s are taken into account individually in the estimation of e.s.d.'s in distances, angles and torsion angles; correlations between e.s.d.'s in cell parameters are only used when they are defined by crystal symmetry. An approximate (isotropic) treatment of cell e.s.d.'s is used for estimating e.s.d.'s involving l.s. planes.
Refinement. Refinement of *F*^2^ against ALL reflections. The weighted *R*-factor *wR* and goodness of fit *S* are based on *F*^2^, conventional *R*-factors *R* are based on *F*, with *F* set to zero for negative *F*^2^. The threshold expression of *F*^2^ > σ(*F*^2^) is used only for calculating *R*-factors(gt) *etc*. and is not relevant to the choice of reflections for refinement. *R*-factors based on *F*^2^ are statistically about twice as large as those based on *F*, and *R*- factors based on ALL data will be even larger.

### Fractional atomic coordinates and isotropic or equivalent isotropic displacement parameters (Å^2^)

**Table:**

	*x*	*y*	*z*	*U*_iso_*/*U*_eq_	
O2	0.81372 (17)	0.37827 (10)	0.59844 (6)	0.0305 (4)	
O1	0.33079 (16)	0.12481 (10)	0.60649 (6)	0.0293 (4)	
O3	0.29982 (17)	0.39411 (11)	0.69152 (7)	0.0340 (4)	
C4	0.7076 (2)	0.22511 (15)	0.57426 (9)	0.0249 (5)	
C2	0.8442 (2)	0.11042 (16)	0.51523 (9)	0.0264 (5)	
H2	0.9313	0.0936	0.4953	0.032*	
C1	0.7206 (2)	0.04620 (15)	0.51579 (8)	0.0240 (5)	
C9	0.4425 (2)	0.27435 (16)	0.63512 (9)	0.0259 (5)	
C5	0.5831 (2)	0.16126 (15)	0.57533 (9)	0.0235 (4)	
C7	0.6948 (2)	0.31407 (15)	0.60336 (9)	0.0260 (5)	
C3	0.8384 (2)	0.19642 (15)	0.54331 (9)	0.0257 (5)	
H3	0.9215	0.2370	0.5422	0.031*	
C10	0.4513 (2)	0.18827 (15)	0.60595 (9)	0.0254 (5)	
C6	0.5934 (2)	0.07262 (14)	0.54638 (9)	0.0243 (5)	
H6	0.5122	0.0305	0.5479	0.029*	
C11	0.5454 (3)	0.43732 (15)	0.65890 (10)	0.0312 (5)	
H11A	0.6029	0.4843	0.6372	0.037*	
H11B	0.5819	0.4371	0.6984	0.037*	
C8	0.5659 (2)	0.33918 (16)	0.63246 (9)	0.0267 (5)	
C13	0.2982 (3)	0.29950 (16)	0.66695 (9)	0.0314 (5)	
H13	0.2144	0.2957	0.6395	0.038*	
C12	0.3790 (3)	0.46387 (17)	0.65792 (10)	0.0354 (6)	
H12	0.3422	0.4622	0.6180	0.043*	
C17	0.3459 (3)	0.56069 (17)	0.68390 (11)	0.0430 (7)	
H17A	0.2397	0.5733	0.6816	0.065*	
H17B	0.3999	0.6093	0.6630	0.065*	
H17C	0.3768	0.5612	0.7237	0.065*	
C15	0.9036 (3)	0.3855 (2)	0.64991 (10)	0.0382 (6)	
H15A	0.8431	0.4102	0.6809	0.057*	
H15B	0.9868	0.4281	0.6430	0.057*	
H15C	0.9411	0.3232	0.6601	0.057*	
C14	0.2395 (3)	0.12913 (18)	0.55544 (10)	0.0382 (6)	
H14A	0.1977	0.1924	0.5516	0.057*	
H14B	0.1594	0.0831	0.5583	0.057*	
H14C	0.3003	0.1148	0.5222	0.057*	
C16	0.2646 (3)	0.23321 (17)	0.71690 (10)	0.0427 (6)	
H16A	0.3452	0.2365	0.7445	0.064*	
H16B	0.2552	0.1686	0.7029	0.064*	
H16C	0.1721	0.2524	0.7350	0.064*	

### Atomic displacement parameters (Å^2^)

**Table:**

	*U*^11^	*U*^22^	*U*^33^	*U*^12^	*U*^13^	*U*^23^
O2	0.0365 (9)	0.0286 (8)	0.0265 (8)	−0.0083 (7)	−0.0003 (7)	0.0009 (7)
O1	0.0278 (8)	0.0357 (8)	0.0244 (8)	−0.0046 (7)	0.0007 (6)	0.0002 (7)
O3	0.0385 (9)	0.0344 (9)	0.0292 (9)	0.0067 (8)	0.0023 (7)	−0.0034 (7)
C4	0.0283 (11)	0.0256 (11)	0.0208 (11)	0.0011 (9)	−0.0019 (8)	0.0030 (9)
C2	0.0239 (10)	0.0329 (12)	0.0224 (12)	−0.0005 (9)	0.0010 (8)	0.0024 (10)
C1	0.0284 (10)	0.0243 (11)	0.0194 (11)	0.0012 (9)	−0.0028 (8)	0.0035 (9)
C9	0.0309 (11)	0.0291 (12)	0.0178 (10)	0.0024 (9)	0.0001 (8)	0.0027 (9)
C5	0.0266 (10)	0.0245 (11)	0.0194 (11)	0.0015 (9)	−0.0014 (8)	0.0034 (9)
C7	0.0318 (11)	0.0236 (11)	0.0226 (11)	−0.0019 (9)	−0.0018 (9)	0.0035 (9)
C3	0.0249 (10)	0.0276 (12)	0.0247 (11)	−0.0038 (9)	−0.0004 (9)	0.0030 (9)
C10	0.0255 (11)	0.0286 (11)	0.0221 (10)	−0.0008 (9)	−0.0006 (8)	0.0050 (10)
C6	0.0255 (10)	0.0256 (11)	0.0218 (11)	−0.0024 (9)	−0.0023 (9)	0.0020 (9)
C11	0.0412 (13)	0.0235 (11)	0.0288 (12)	0.0012 (10)	0.0022 (10)	0.0005 (10)
C8	0.0346 (11)	0.0261 (11)	0.0193 (11)	0.0031 (9)	−0.0015 (9)	0.0048 (9)
C13	0.0316 (12)	0.0336 (13)	0.0289 (12)	0.0051 (10)	0.0021 (9)	−0.0043 (10)
C12	0.0504 (14)	0.0315 (13)	0.0243 (12)	0.0103 (11)	−0.0012 (10)	0.0012 (11)
C17	0.0602 (16)	0.0371 (14)	0.0318 (14)	0.0160 (12)	−0.0048 (12)	−0.0012 (12)
C15	0.0364 (13)	0.0429 (14)	0.0354 (14)	−0.0068 (11)	−0.0038 (11)	−0.0004 (12)
C14	0.0303 (12)	0.0498 (16)	0.0346 (14)	−0.0090 (11)	−0.0032 (10)	0.0045 (12)
C16	0.0480 (14)	0.0448 (15)	0.0354 (13)	−0.0002 (12)	0.0158 (12)	−0.0041 (12)

### Geometric parameters (Å, °)

**Table:**

O2—C7	1.388 (2)	C6—H6	0.9300
O2—C15	1.436 (3)	C11—C8	1.509 (3)
O1—C10	1.388 (2)	C11—C12	1.523 (3)
O1—C14	1.433 (3)	C11—H11A	0.9700
O3—C12	1.429 (3)	C11—H11B	0.9700
O3—C13	1.435 (3)	C13—C16	1.508 (3)
C4—C7	1.415 (3)	C13—H13	0.9800
C4—C3	1.421 (3)	C12—C17	1.505 (3)
C4—C5	1.419 (3)	C12—H12	0.9800
C2—C3	1.364 (3)	C17—H17A	0.9600
C2—C1	1.416 (3)	C17—H17B	0.9600
C2—H2	0.9300	C17—H17C	0.9600
C1—C6	1.382 (3)	C15—H15A	0.9600
C1—C1^i^	1.480 (4)	C15—H15B	0.9600
C9—C10	1.378 (3)	C15—H15C	0.9600
C9—C8	1.421 (3)	C14—H14A	0.9600
C9—C13	1.518 (3)	C14—H14B	0.9600
C5—C6	1.408 (3)	C14—H14C	0.9600
C5—C10	1.418 (3)	C16—H16A	0.9600
C7—C8	1.373 (3)	C16—H16B	0.9600
C3—H3	0.9300	C16—H16C	0.9600
			
C7—O2—C15	113.58 (16)	C9—C8—C11	117.76 (19)
C10—O1—C14	113.67 (15)	O3—C13—C16	105.14 (17)
C12—O3—C13	114.47 (16)	O3—C13—C9	113.29 (18)
C7—C4—C3	123.47 (19)	C16—C13—C9	113.37 (19)
C7—C4—C5	118.55 (18)	O3—C13—H13	108.3
C3—C4—C5	117.97 (18)	C16—C13—H13	108.3
C3—C2—C1	121.43 (18)	C9—C13—H13	108.3
C3—C2—H2	119.3	O3—C12—C17	107.21 (19)
C1—C2—H2	119.3	O3—C12—C11	107.70 (18)
C6—C1—C2	118.01 (18)	C17—C12—C11	113.7 (2)
C6—C1—C1^i^	118.94 (14)	O3—C12—H12	109.4
C2—C1—C1^i^	123.03 (14)	C17—C12—H12	109.4
C10—C9—C8	119.23 (18)	C11—C12—H12	109.4
C10—C9—C13	119.07 (19)	C12—C17—H17A	109.5
C8—C9—C13	121.64 (19)	C12—C17—H17B	109.5
C6—C5—C10	121.61 (19)	H17A—C17—H17B	109.5
C6—C5—C4	119.37 (18)	C12—C17—H17C	109.5
C10—C5—C4	119.02 (18)	H17A—C17—H17C	109.5
C8—C7—O2	120.66 (18)	H17B—C17—H17C	109.5
C8—C7—C4	121.54 (19)	O2—C15—H15A	109.5
O2—C7—C4	117.67 (18)	O2—C15—H15B	109.5
C2—C3—C4	121.19 (19)	H15A—C15—H15B	109.5
C2—C3—H3	119.4	O2—C15—H15C	109.5
C4—C3—H3	119.4	H15A—C15—H15C	109.5
C9—C10—O1	120.42 (18)	H15B—C15—H15C	109.5
C9—C10—C5	121.46 (19)	O1—C14—H14A	109.5
O1—C10—C5	118.11 (18)	O1—C14—H14B	109.5
C1—C6—C5	122.02 (19)	H14A—C14—H14B	109.5
C1—C6—H6	119.0	O1—C14—H14C	109.5
C5—C6—H6	119.0	H14A—C14—H14C	109.5
C8—C11—C12	109.3 (2)	H14B—C14—H14C	109.5
C8—C11—H11A	109.8	C13—C16—H16A	109.5
C12—C11—H11A	109.8	C13—C16—H16B	109.5
C8—C11—H11B	109.8	H16A—C16—H16B	109.5
C12—C11—H11B	109.8	C13—C16—H16C	109.5
H11A—C11—H11B	108.3	H16A—C16—H16C	109.5
C7—C8—C9	120.13 (19)	H16B—C16—H16C	109.5
C7—C8—C11	121.99 (19)		

Symmetry codes: (i) *x*, −*y*, −*z*+1.
